# Performance of the Afferent Limb of Rapid Response Systems in Managing Deteriorating Patients: A Systematic Review

**DOI:** 10.1155/2019/6902420

**Published:** 2019-10-30

**Authors:** Marcello Difonzo

**Affiliations:** ^1^Degree in Nursing, School of Medicine, University of Bari Aldo Moro, Bari, Italy; ^2^Intensive Care Unit, Di Venere Teaching Hospital, Bari, Italy

## Abstract

**Introduction:**

The clinical components of the rapid response system (RRS) are the afferent limb, to ensure identification of in-hospital patients who deteriorate and activation of a response, and the efferent limb, to provide the response. This review aims to evaluate the factors that influence the performance of the afferent limb in managing deteriorating ward patients and their effects on patient outcomes.

**Methods:**

A systematic review was performed for the years 1995–2017 by employing five electronic databases. Articles were included assessing the ability of the ward staffs to monitor, recognize, and escalate care to patient deterioration. The findings were summarized using a narrative approach.

**Results:**

Thirty-one studies met the inclusion criteria. The analysis revealed major themes enclosing several factors affecting management of patients having sudden deterioration. The monitoring and recognition process was conditioned by the lack of recording of physiological parameters, the influence of facilitators, including staff education and training, and barriers, including human and environmental factors, and poor compliance with the calling criteria. The escalation of care process highlighted the influence of cultural barriers and personal judgment on RRS activation. Mainly, delayed team calls were factors strongly associated with the increased risk of unplanned admissions to the intensive care unit and length of stay, hospital length of stay and mortality, and 30-day mortality.

**Conclusions:**

A combination of factors affects the timely identification and response to sudden deterioration by general ward staffs, leading to suboptimal care of patients, delayed or failed activation of RRS teams, and increased risks of worsening outcomes. The research efforts and clinical involvement to improve the governance of the factors limiting the performance of the afferent limb may ensure proper management of hospitalized patients showing physiological deterioration.

## 1. Introduction

On general hospital wards, the timely treatment of patient deterioration before the onset of serious adverse events (SAEs) is achievable by alerting emergency teams of critical care clinicians. The Medical Emergency Team (MET) has been adopted, the first of these teams, in 1989 at the Liverpool Hospital in Sydney, Australia [1, 2], to supplement or replace the Cardiac Arrest Team (CAT) [3]. Afterward, the Rapid Response Team (RRT) in the United States of America (USA), the Critical Care Outreach Service (CCOS) in the United Kingdom (UK), and the Critical Care Response Team (CCRT) in Canada [4–7] have been proposed. In 2006, the concept of the Rapid Response System (RRS) has merged the previous models of emergency teams by integrating four components [8, 9]. The afferent limb includes ward physicians and nurses to identify at-risk patients and to trigger a response based on the calling criteria. The efferent limb ensures the response with emergency teams of critical care doctors and nurses. The last two are the administrative limb, for coordinating all system components, and the quality improvement limb, for improving the function of the RRS [8, 9]. The calling criteria include vital signs, physiological parameters, and the level of consciousness, the objective criteria, and the staff “worried” criterion or concern about the patient, the subjective criterion. These tools are named the physiological track and trigger warning systems (TTSs) and consist of single-parameter systems, multiple-parameter systems, aggregate weighted scoring systems, and combination systems [10, 11].

The RRS has been designed to detect and respond to deteriorating patients outside the intensive care unit (ICU) [8, 9]. The concept of patient deterioration has been first emphasized by Schein et al. [12] by suggesting that derangement of clinical signs often anticipates cardiopulmonary arrest. Franklin and Mathew [13] have reported patient deterioration documented by physicians or nurses within 6 hours of cardiac arrest, emphasizing the failure to respond to cardiopulmonary, neurological, or respiratory deterioration. Jones et al. [14] have described a deteriorating patient as one with an increased risk of morbidity, organ dysfunction, protracted hospital stay, disability, or death. Mostly, the early detection and treatment of patients at risk of clinical deterioration improve their outcomes [15]. A prospective cohort study involving 48 hospitals in the UK has shown high 90-day mortality among deteriorating ward patients, whereas early ICU admission within 4 hours of the assessment has strongly reduced mortality [16].

**Figure 1:**
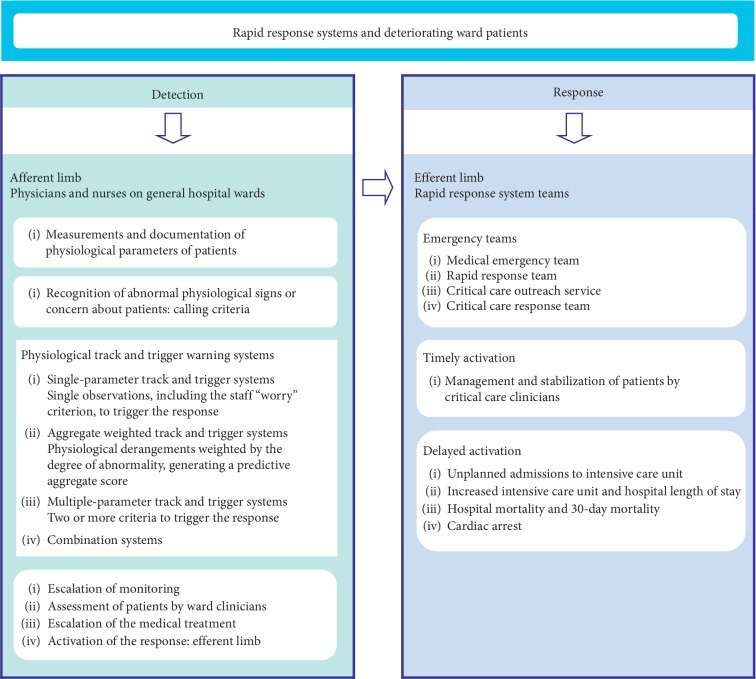
Rapid response systems and management of deteriorating ward patients.

Clinical performance measurement, in a health system intervention, involves how measures are created, how they are implemented, and the evidence of their potential benefits and harms [17]. The RRS aims to reduce SAEs including cardiac arrest, unplanned admissions to the ICU, and death [8, 9]; however, the effectiveness of RRSs in improving patient outcomes remains controversial [18–20]. Regardless, evidence from recent meta-analyses [21–23] has suggested that implementation of RRSs has substantially reduced non-ICU cardiac arrests, hospital mortality, and unexpected mortality in the adult population without an evident effect on ICU admission rates. Afferent limb failure (ALF) has been indicated as the presence of documented MET calling criteria without a MET call before an in-hospital serious event [24] and has been proposed as a performance measure of RRSs [25]. Practically, the performance of the afferent limb is difficult to evaluate. Winters et al. [26] have indicated facilitators and barriers to system implementation, such as acceptance and leadership of the RRS, rates of calling the RRS, and trigger mechanisms. Besides, different factors may encourage or inhibit the effective use of the MET system by ward nurses [27]. The dynamic of the afferent limb relies on the interaction and collaboration between physicians and nurses with different clinical skills. The activity of these clinicians involves sequential passages: monitoring of vital signs and physiologic parameters, recognizing of patient deterioration, implementation of the treatment for at-risk patients, and the request for help with activation of RRS teams (Figure 1). This review aims to evaluate the factors that influence the performance of the afferent limb, affecting the ability to monitor, recognize, and escalate care to deteriorating ward patients, and their effects on patient outcomes.

## 2. Methods

### 2.1. Study Design

A systematic review methodology was adopted by following the Preferred Reporting Items for Systematic Reviews and Meta-Analyses Protocol (PRISMA) tool [28] ([Supplementary-material supplementary-material-1] in Supplementary Materials).

### 2.2. Eligibility Criteria and Study Selection

The eligibility criteria to include studies agreed with the SPIDER (Sample, Phenomenon of Interest, Design, Evaluation, Research type) tool [29] ([Supplementary-material supplementary-material-1] in Supplementary Materials). The review included primary research studies on factors influencing the ability to monitor, recognize, and escalate care to patient deterioration; assessing the effects of these factors on patient outcomes; conducted on general wards; involving adult patients (>18 years of age), ward physicians, or ward nurses in acute hospitals; and published in English between 1995 and 2017. Lee et al. [1] first outlined, in 1995, the concept of the RRS as critical care clinicians responding to deteriorating patients outside the ICU, so the present research included papers following this work. The exclusion criteria included editorials, commentaries, opinion papers, reviews, pediatric patients, and non-English languages.

### 2.3. Search Strategy

A systematic search covered the literature published between 1 January 1995 and 31 December 2017 by using five electronic databases. The web search contemplated the bibliographic databases CINAHL, Medline, ScienceDirect, Scopus, and the search engine Google Scholar. In addition, the reference lists of the selected papers were examined to identify further relevant studies. The search involved different keywords combined with the Boolean logic. The following terms were included: deteriorating patients, rapid response systems, medical emergency team, rapid response team, critical care outreach service, critical care response team, patient monitoring, patient recognizing, escalation of care, and general wards ([Supplementary-material supplementary-material-1] in Supplementary Materials).

### 2.4. Quality Assessment

All included papers were evaluated by the Quality Assessment Tool for Studies with Diverse Designs (QATSDD) instrument [30], which allows the quality assessment of studies with different methodological designs, such as quantitative, qualitative, and mixed methods studies. The QATSDD includes 14–16 items with a three-point scale by assigning a score to each study.

### 2.5. Data Extraction and Synthesis

The records identified by the search were imported into the reference management software Zotero. After screening the title and the abstract of each article, the entire manuscripts suitable for inclusion were read. One single researcher extracted the data according to predefined criteria, including authors, year and country, aim and design, sample and outcome measures, and findings. All the eligible studies were assessed for their quality. The reviewed studies presented heterogeneous designs. Therefore, the synthesis of the data followed the narrative synthesis proposed by Popay et al. [31]. This approach to a systematic review uses words and texts instead of numbers to summarize and explain the results of the synthesis.

## 3. Results

### 3.1. Study Selection

**Figure 2:**
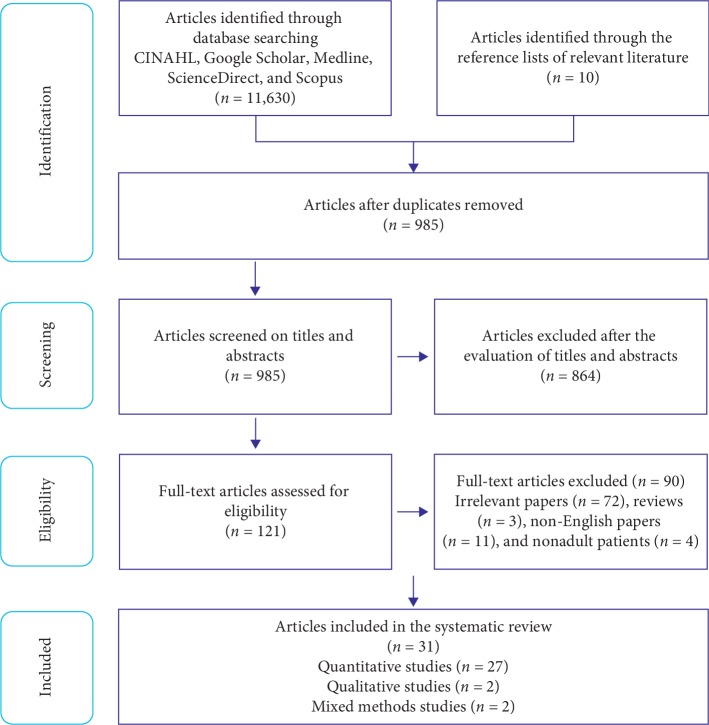
The PRISMA diagram of the study search strategy.

The search from all sources generated 11,640 articles. The removal of duplicates and nonrelevant papers produced 121 potential studies. Following a full-text review, 31 articles were included (Figure 2).

### 3.2. Study Characteristics

The studies represented 11 countries with the majority originated from Australia (*n*=13), followed by the Netherlands (*n*=4), the UK (*n*=3), the USA (*n*=3), and Canada (*n*=2) with one study each from Brazil, Denmark, Finland, Greece, Italy, and Spain. The study setting included community, teaching, and university hospitals (*n*=30) and one simulation scenario (27 quantitative, two qualitative, and two mixed methods studies). The sample size ranged from 14 ward staff members to 125,132 patients of the MERIT trial. The population included patients, physicians, and nurses on general hospital wards ([Supplementary-material supplementary-material-1] in Supplementary Materials).

### 3.3. Study Quality Assessment

The average QATSDD score was 53.34% with a range between 40.47% and 73.8%. The overall quality of the studies was moderate to strong. Few studies reported the evidence of the sample size, the target group of a reasonable size, and relevance of the user involvement in the design. The statistical assessment of validity and reliability was poor. Most studies described adequately the research settings and procedures for data collection and recruitment.

### 3.4. Synthesis of Results

Previous studies identified three stages that affect the efficiency of the afferent limb, such as monitoring, recognizing, and escalating care to patient deterioration [25, 32]. These findings were consistent with the objectives of the present review. Therefore, the reported studies [33–63] followed these stages and were presented in the table format (Tables 1–3) and in the text format. Besides, the common areas within the studies were categorized into themes relevant to the objectives of the review.

### 3.5. Monitoring Deteriorating Patients

Eleven papers investigated monitoring of deteriorating patients, treating the following themes: lack of recording, poor documentation of the respiratory rate, and influence of facilitators and barriers. Overall, the lack of recording of physiological measurements was observed in all reported papers [33–43]. In the MERIT trial [33], monitoring, recording, and responding to vital signs changes were lacking. Indeed, MET calls were documented in only 30% of patients with the calling criteria before ICU admissions, and several patients with documented MET criteria were identified less than 15 minutes before an adverse event. McGain et al. [34] reported complete documentation of medical and nursing review and vital signs in only 17% of patients after major surgery in five hospitals. Similarly, Chen et al. [36] showed the lack of at least one vital sign in 77% of patients with adverse events. Ludikhuize et al. [37] documented the complete recording of vital signs in 30–66% of assessments. Differently, Pantazopoulos et al. [38] described documentation of vital signs every 6 hours performed only by 43% of nurses. Tirkkonen et al. [39] assessed suboptimal documentation of vital signs in normal wards versus wards with automated noninvasive monitoring (74% vs. 96%, *p* < 0.001), especially relevant for the respiratory rate (17% vs. 75%, *p* < 0.001). Besides, ALF was more common among patients with automated versus traditional monitoring (81% vs. 53%, *p* < 0.001), emphasizing the role of timely interventions to obtain benefits from extensive monitoring, and was independently related to increased hospital mortality. The work of Cardona-Morrell et al. [41] evidenced vital signs assessments in 52% of nurse-patient interactions. They reported five vital signs (blood pressure, heart rate, respiratory rate, temperature, and SpO_2_) monitored on average in 21% of cases and only in 6% of patients in a surgical ward. Lastly, Considine et al. [42] reported evidence of abnormal physiological parameters in 79.8% of patients, but only in 19.7% of them were abnormalities documented.

Seven papers indicated poor documentation of the respiratory rate. Indeed, this vital sign was the less documented, the recording rate was 14–17% [34–36, 38, 39, 43], and only one report indicated a higher recording rate (30–66%) [37].

Four papers underlined the role of facilitators and barriers [36, 38, 40, 43]. A post hoc study [36] found that missing documentation of vital signs was significantly reduced with the introduction of the MET system. Pantazopoulos et al. [38] observed a high level of judgment, including MET activation, in nurses graduated from a 4-year against a 2-year degree course; besides, those trained with Basic Life Support and Advanced Life Support courses identified and managed cardiac or respiratory emergencies better. Ludikhuize et al. [40] reported better calculations of Modified Early Warning Score by nurses in protocolized wards (three times daily measurements of vital signs) versus control wards (70% vs. 2%, *p* < 0.001). Besides, compliance with measurements of vital signs was better in protocolized wards versus the control group (68% vs. 4%) with more reliable RRT activation. Lastly, Smith and Aitken [43] identified factors interfering with monitoring and escalation of clinical deterioration, including the lack of monitoring equipment, the workload, interactions and conflicts between the staff, and interactions with patients.

### 3.6. Recognizing Deteriorating Patients

Six papers described recognition of deteriorating patients, treating the following themes: compliance with the calling criteria and impact of communication. Five papers explored the compliance with the Early Warning Scores (EWSs), reporting poor adherence with the protocol [44–48]. The EWSs utilize deviation of multiple parameters from the normal ones, weighted and converted into a single score, with higher risks of clinical deterioration for higher scores. The Early Warning Scoring system was developed in 1997 by Morgan et al. [64]. Later, it was proposed the Modified Early Warning Score (MEWS) [65] and the National Early Warning Score (NEWS) adopted across the National Health Service (NHS) in the UK [66]. In this review, Donohue and Endacott [44] underlined the use of a visual evalution by comparing the patient's clinical condition over time by nurses who used the MEWS to quantify deterioration after recognition of the clinical instability. A simulation study [45] reported the MEWS correctly determined by only 11% of the trained nurses; however, the trained group assessed the patient immediately (77% vs. 58%, *p* = 0.056) and measured the respiratory rate twice as frequently compared to the nontrained nurses (53% vs. 25%, *p* = 0.025). Kolic et al. found [46] an incorrect calculation of the NEWS in 18.9% of patients with an inadequate clinical response in 25.9% of cases and scoring errors more frequently with higher NEW scores. The study by van Galen et al. [47] showed vital signs monitoring performed as agreed with the doctors in only 41% of patients; besides, 43% of measurements had a critical MEWS (≥3) 48 hours before ICU admissions, but only 1% of measurements had a correct calculation. Lastly, Petersen et al. [48] documented low adherence to the EWS monitoring frequency often during busy periods and at night, low rate calls of the junior doctors for patients with a high EWS, and barriers for negative feelings toward the MET system by nurses.

One paper highlighted the role of communication between clinicians. Wong et al. [49] reported messages between nurses and physicians with information on the calling criteria before the ICU transfer in about 39% of patients, but only 45% of messages included two or more vital signs.

### 3.7. Escalating Care to Deteriorating Patients

Fourteen papers explored escalation of care to deteriorating patients, treating the following themes: influence of cultural barriers and personal judgment, delayed team calls, and effects of delays on clinical outcomes. Four papers identified cultural barriers preventing timely escalation of care [50, 52, 55, 59]. Jones et al. [50] described the traditional approach of initially calling ward doctors by nurses (72%) who would call the MET for a patient they were worried, even with normal vital signs (56%). A survey of Canadian nurses [52] underlined the fear of criticism (15.4%) and the hierarchical model of alerting the responsible physician before the MET call (75.9%), also if respondents (48%) would activate the MET system for a patient they were concerned about. Local sociocultural factors and intraprofessional hierarchies were other barriers to RRS activation [55]. Radeschi et al. [59] indicated the covering physician as the major barriers to MET activation for nurses (62%); besides, the reluctance to the MET call in a patient fulfilling the calling criteria (21%) was more frequent for nurses than for doctors.

Two papers identified the impact of personal judgment on team activation. Shearer et al. [55] reported missing RRS calls because the bedside staff believed the clinical situation was under control (51.8%) or RRS activation was not necessary for staff experience with patient deterioration (14%). Davies et al. [57] showed low adherence (25%) to six criteria for RRT activation related to different importance given by ward staffs to the different calling criteria.

Nine papers reported delayed or missed MET calls from 21.4% to 57% of patients who had documented calling criteria with delayed team alerts ranging from 15 minutes to 24 hours [51, 53–56, 58, 60–62]. Eight papers assessed the patient outcomes related to delayed calls [53, 54, 56, 58, 60–63]. Calzavacca et al. [53] found less delayed MET activation, 5 years after RRS implementation, in a recent cohort versus a control cohort of patients (22% vs. 40.3%, *p* < 0.001). They reported delayed MET activation associated with the increased risk of unplanned ICU admission (OR 1.79, 95% CI 1.33–2.93, *p* = 0.003) and hospital mortality (OR 2.18, 95% CI 1.42–3.33, *p* < 0.001). Similarly, Trinkle and Flabouris [54] documented 22.8% of ALF in patients with adverse events that, compared to patients without ALF, presented a higher risk of unscheduled ICU admissions (34.4 vs. 22.5%, *p* = 0.01) and hospital mortality (52.5% vs. 31.9%, *p* = 0.03) through multiple, as opposed to single, time periods. Boniatti et al. [56] found 21.4% of delayed calls, significantly higher for physicians versus nurses (29.2% vs. 17.6%, *p* < 0.001); besides, 30-day mortality after the MET review was higher in patients with delayed compared to timely MET activation (61.8% vs. 41.9%, *p* < 0.001). A post hoc analysis of the MERIT study [58] reported delayed calls in 30.2% of patients with the increased risk of unplanned ICU admissions (OR 1.56, 95% CI 1.23–2.04, *p* ≤ 0.001) and death (OR 1.79, 95% CI 1.43–2.27, *p* < 0.001) in the control and MET hospitals. Barwise et al. [60] described delayed RRT activation in 57% of patients associated with higher hospital mortality (15% vs. 8%, OR 1.6, *p* = 0.005), 30-day mortality (20% vs. 13%, OR 1.4, *p* = 0.02), and hospital length of stay (LOS) (7 vs. 6 days, relative prolongation 1.10, *p* = 0.02) compared to the no-delay group. Castano-Avila et al. [61] reported delayed alerts (41.25%) in patients admitted to the ICU. These admissions showed a significantly higher APACHE II score, SAPS II score, MODS rate, and nonsignificant longer length of ICU stay. Gupta et al. [62] showed 24.6% of delayed rapid response calls related to the increase of in-hospital mortality (34.7% vs. 21.2%, *p* = 0.001) and longer hospitalization (11.6 vs. 8.4 days, *p* = 0.036). Lastly, Sprogis et al. [63] underlined a high frequency of delayed escalation of care by ward clinicians with 58% of patients without a documented response by nurses to first urgent clinical review criteria, and 12% of hospital mortality for patients requiring MET activation.

## 4. Discussion

This review explores the literature on different aspects interfering with the performance of the afferent limb of RRSs. The research identifies several factors enabling or inhibiting the ability of ward staffs to monitor and record physiologic parameters, recognize physiological deterioration, and escalate care to unexpectedly deteriorating patients.

Monitoring of deteriorating patients in this review emphasized the lack of recording since measurements and documentation of physiological parameters had high variability, and they were rarely recorded and often undocumented [33–43]. The literature suggests the need for more reliable monitoring of vital signs. In an ICU, patients have continuous monitoring of multiple physiological parameters. In a general ward, monitoring may be intermittent or continuous, manual or automated, and often includes only traditional vital signs. Intermittent monitoring is not always adequate to highlight timely changes in vital signs. Nonetheless, evidence of effectiveness was insufficient to recommend continuous vital signs monitoring as routine practice in general wards [67]. The monitoring process required both a correct interpretation of physiologic disorders and an adequate response to these observations [68]. The optimal frequency of vital sign measurements to increase the likelihood of detecting clinical deterioration is unclear. In the UK, the minimum frequency of monitoring should be at least every 12 hours [66]. An Australian consensus statement suggested the frequency of observation at least once per 8-hour shift [69], while another statement suggested the intermittent assessment of vital signs should occur every 12 hours or preferably every 6 hours [68]. The trends of vital signs compared to the value of vital signs alone substantially improved the accuracy of deterioration detection and were independent predictors of critical illness in ward patients [70]. Basic biochemistry and hematology results were other relevant signs for early detection of the patient in crisis [68]. Spanish papers emphasized an alert system to avoid emergency ICU admissions with early identification of patient deterioration based on laboratory tests selected for organ failure. The authors reported a decrease in the ICU mortality rate after admissions of at-risk patients by evaluating the alteration of these laboratory tests [71] and by extending this evaluation to weekends and public holidays [72]. Ward nurses were indicated as responsible for the assessment, recording, and documentation of vital signs [73]; however, evidence indicates their poor compliance with vital signs monitoring. Chua et al. [74] described the incomplete vital signs monitoring and interpretation by nurses for the excessive workload and the lack of recognition of the importance of vital signs, particularly the respiratory rate. Similarly, Mok et al. [75] explored nurses' attitudes revealing the limited understanding of key indicators of deterioration. Furthermore, nurses indicated monitoring of vital signs as being time consuming, overwhelming, and unnecessary for patients with stable conditions [74, 75].

Poor documentation of the respiratory rate in the reviewed studies underlined frequent and repeated omissions of this measurement during vital signs monitoring [34–36, 38, 43]. Comparable findings are demonstrated by other researchers. The respiratory rate was the most commonly undocumented observation with the missing rate ranging from 0.8% to 61.8% of patients in different hospitals [76]. Elliott [77] reported poor understanding regarding the importance of the respiratory rate as vital signs by nurses for inadequate knowledge of the respiratory rate assessment, nurses' perception of the patient's acuity, and the lack of time. Moreover, the respiratory rate was claimed as an early indicator of serious illness, such as shock, sepsis, and respiratory insufficiency, since its increase reflects hypoxia and metabolic acidosis [78].

Facilitators and barriers to the monitoring process highlighted different issues in the selected studies. RRS implementation substantially increased the vital signs recording [36], while higher degrees and training courses helped nurses to better identify emergencies and patient deterioration [38]. Standardized measurements of the vital signs and MEWS allowed more efficient activation of ward physicians and RRS teams by nurses [40]. The failure to monitor was correlated to the lack of monitoring equipment and human and environmental interfering factors [43]. Published studies identify comparable results. The nurses attending a MET training session showed a greater intention to call the MET and correctly identified most MET activation criteria [79]. Moreover, strategies as educational development and modification of clinical processes of patient monitoring could improve recognizing and managing of deteriorating patients by nurses [74].

Recognition of deteriorating patients in this review suggested poor compliance with the EWS protocol. Indeed, there was a low percentage of correct measurements, particularly with high EWS ranges, worsening of clinical responses at weekends, increased mortality with incorrect responses, and low agreement with the monitoring frequency, particularly during busy periods and at night [44, 46–48]. Furthermore, the favorable effects of the training to improve compliance with the EWS were also described [45]. Previous papers suggest similar issues. The EWSs had a good predictive value for patient deterioration and improve patient outcomes, but inaccurate recordings or inappropriate reactions to abnormal scores could reduce these benefits [80]. Besides, the efficiency of the EWSs depended on the patient cohort, facilities available, and the staff training and attitude [81]. Regardless, the EWSs could not replace clinical judgment and clinical skills [80, 81].

Poor communication between nurses and physicians in the reviewed studies was expressed by the low quality of critical messages on patient deterioration and the positive relationship between the quality of messages and hospital survival [49]. Similarly, a previous study indicated the role of inadequate communication between clinicians in management of patient deterioration [82].

Escalation of care to deteriorating patients in the present review underlined the effects of cultural barriers and personal judgment on the response. Cultural barriers as the nurses' hierarchical approach, intraprofessional hierarchies between the ward clinicians, and reluctance to call the MET prevented timely response activation by the ward staffs [50, 52, 55, 59]. Subjective judgment induced a failure to respond when the staff judged the clinical situation to be under control in the ward and poor compliance toward RRS activation for low adherence to the calling criteria [55, 57]. Analogously, the previous study by Odell et al. [82] suggested that nurses used intuitive judgment to assess deterioration, using vital signs to confirm their findings. Another study [83] indicated hierarchical organization and poor interprofessional communication as causes of delayed escalation, underlining also the role of the high workload and overconfidence.

Delayed team calls in the reviewed studies involved several patients (21-57%) who fulfilled the calling criteria for emergency teams [51, 53–56, 58, 60–62]. Mostly, there was a strong increase in the risks of unplanned ICU admissions [53, 54, 58], hospital LOS [60, 62], hospital mortality [53, 54, 58, 60, 62], 30-day mortality [56, 60], and prolonged ICU LOS [61] related to delayed or missed alerts. The main trigger for timely MET calls was the concern about the patient for nurses, and delayed calls were higher for physicians than for nurses [56]. Similar findings are underlined by previous studies. A multicenter study [84] in 17 ICUs demonstrated 71% of admissions with unnecessary delays for organizational issues rather than patient-related problems. Similarly, Sankey et al. [85] reported 64.6% of delayed escalation of care greater than 4 hours in 793 patients before the ICU transfer and a substantial increase in in-hospital mortality for delays over 12 hours. The reasons for delayed team calls are linked to the difference between the diverse calling criteria used and the role of the staff “worried” criterion to activate the MET system, which involves ward nurses much more frequently than doctors. Santiano et al. [86] reported that the “worried” criterion was the most frequent reason for MET calls (29% of 3,194 team calls) in six acute hospitals. They also underlined that this subjective criterion often relied on clinical intuition and judgment of ward nurses. Similarly, Mezzaroba et al. [87] confirmed as the most frequent reason (37.7%) for emergency team activation was the ward team seriously concerned about the patient's clinical instability. Furthermore, the subjective worry or concern criterion by nurses was considered relevant in the early recognition and treatment of deteriorating patients [88].

## 5. Limitations

This review presents several weaknesses. The clinical performance of the afferent limb must consider the differences in warning tools, activation thresholds, and team compositions, doctors, nurses, or other clinicians, physician-led versus nurse-led. Second, the heterogeneity of interventions, study designs, and populations precluded a meta-analysis. Last, one single researcher performed the present review. The credibility of a systematic review may be limited by inappropriate eligibility criteria, the inadequate literature search, or the failure to optimally synthesize results [89]. Moreover, data extraction by two independent reviewers should be used to reduce errors [90]. Nonetheless, a recent paper [91] reported the great prevalence of extraction errors in systematic reviews, although these errors seem to have only a moderate impact on the results and conclusion of the reviews. This research, conducted by one single reviewer, clearly adheres to the protocol, the inclusion and exclusion criteria, the literature search, and the synthesis of results by increasing the transparency and credibility of the process.

## 6. Conclusions

The bedside treatment of patient deterioration on general wards is a complex issue involving physicians and nurses with different expertise. A combination of factors affects the timely identification and response to sudden deterioration by general ward staffs, leading to suboptimal care of patients, delayed or failed activation of RRS teams, and increased risks of worsening outcomes. The research efforts and clinical involvement to improve the governance of the factors limiting the performance of the afferent limb may ensure proper management of hospitalized patients showing physiological deterioration.

## Figures and Tables

**Table 1:** Summary of relevant studies on monitoring deteriorating patients.

Year Authors Country	Aim Design	Sample Outcome measures	Findings
2005 Hillman et al. [33] Australia	To investigate the effectiveness of the MET system in reducing the incidence of cardiac arrests, unplanned admissions to ICU, and deaths Cluster randomized controlled trial in 23 hospitals	MERIT (medical early response, intervention, and therapy) study: 11 hospitals with the CAT (56,756 patients), 12 hospitals with the MET system (68,376 patients) Cardiac arrests, unplanned ICU admissions, or unexpected deaths	(i) No substantial difference in the incidence of cardiac arrests, unplanned ICU admissions, or unexpected deaths (ii) The MET was involved in only 30% of patients with the calling criteria before ICU admissions (iii) Several cases of cardiac arrests (53%, 46%), unplanned ICU admissions (21%, 36%), and unexpected deaths (34%, 25%) were identified less than 15 min before the event in the control and MET hospitals, respectively

2008 McGain et al. [34] Australia	To describe factors associated with incomplete postoperative documentation of vital signs Retrospective observational study in five hospitals	211 adult patients after major surgery Documentation of medical and nursing reviews and vital signs	(i) In the first 3 days after surgery, 17% of patient records had complete documentation of vital signs (BP, HR, RR, T°, and SpO_2_) and medical and nursing reviews (ii) The most undocumented observation was the RR (15.4%)

2008 Van Leuvan and Mitchell [35] Australia	To determine the frequency of vital sign measurements and differences in the frequency between specific vital signs Retrospective observational study in one tertiary hospital	1,597 unique vital signs recorded in 62 patients Readings of vital signs from all patient charts	(i) Documentation of vital signs was significantly lower for RR (1.0 reading/day) vs. BP (5.0 readings/day), HR (4.4 readings/day), and T° (4.2 readings/day), *p* < 0.001 for all comparisons

2009 Chen et al. [36] Australia	To examine the effect of the MET system introduction on the documentation rate of vital signs during the MERIT study Post hoc analysis in 23 hospitals	Vital signs (HR, RR, and SBP) 15 min–24 h before an adverse event (cardiac arrest, death, or unexpected ICU admission) or an emergency team call Association between undocumented vital signs, hospital characteristics, and MET allocation	(i) The lack of at least one vital sign in 77% of patients with adverse events (ii) The RR was the lowest documented sign in the control and MET hospitals and was three times missing than the HR and SBP (iii) The MET system improved documentation of the RR and SBP before the emergency team review (*p* < 0.01) and documentation over time (*p* < 0.01)

2012 Ludikhuize et al. [37] Netherlands	To describe measurements and documentation of vital signs and usefulness of the MEWS in detecting deteriorating patients by nurses Retrospective observational study in one university hospital	204 patients in general wards with SAEs (2,688 measures of one or more vital signs 48 h before the event) All documented vital parameters	(i) 81% of patients had a MEWS ≥3 at least once 48 h before the event (cardiopulmonary arrest, unplanned ICU admission, unexpected death, and emergency surgery) (ii) Even with the MEWS ≥3, recordings of vital signs were incomplete: RR, diuresis, and SpO_2_ were documented in only 30–66% of assessments

2012 Pantazopoulos et al. [38] Greece	To test the relationship between nurse demographics and correct identification of clinical situations warranting specific actions and MET activation Cross-sectional survey in one tertiary hospital	94 nurses in general wards Factors influencing MET activation by nurses	(i) Only 43% of nurses recorded vital signs every 6 h (ii) RR and GCS were the less recorded vital signs (iii) Nurses with a 4-year educational course identified a higher rate of emergencies requiring MET activation; those with training in BLS and ALS courses showed better management of cardiac or respiratory emergencies

2013 Tirkkonen et al. [39] Finland	To study the factors associated with delayed MET activation and increased hospital mortality Prospective observational study in one tertiary hospital	A cohort of 569 MET reviews for 458 patients with 5.9% of general ward beds equipped with automatic noninvasive monitoring of vital functions Documentation of vital signs and vital dysfunctions 6 h before a MET call with reference to automated patient monitoring	(i) Vital signs were more frequently documented in patients with automated monitoring vs. normal monitoring (96% vs. 74%, *p* < 0.001) (ii) The RR was alarmingly low (75% vs. 17%, *p* < 0.001) 0–6 h before MET activation (iii) ALF occurred more often among automated monitored vs. normal monitored patients (81% vs. 53%, *p* < 0.001)

2014 Ludikhuize et al. [40] Netherlands	To study the effect of three times daily measurements (protocolized group) of the MEWS vs. measurements clinically indicated (control group) on implementation of the RRS A quasi–experimental study in one university hospital	Sample of patients in 10 protocolized wards and in 8 control wards (372 vs. 432, respectively) Measurements in patients in protocolized and in control wards (3,585 vs. 3,013, respectively)	(i) Nurses estimated the MEWS from vital signs in 70% (2513/3,585) of patients in the protocolized wards vs. 2% (65/3,013) in the control group (*p* < 0.001) (ii) Compliance with the measurement regime (≥3 times per day) was 68% (819/1,205), measurements in the control group were 4% (47/1,232) only (iii) In protocolized wards, there were twice as much RRT calls per admission

2016 Cardona-Morrell et al. [41] Australia	To establish vital signs monitoring practices of nurses and adherence to the health service protocol Prospective observational study in one teaching hospital	42 general ward nurses with 441 patient interactions Vital signs monitoring (HR, BP, RR, T°, SpO_2_, level of consciousness, urine output, and pain)	(i) Vital signs were assessed in 52% (229/441) of interactions (ii) The minimum five measures (BP, HR, RR, T°, and SpO_2_) were taken in 6–21% of instances of vital signs monitoring

2016 Considine et al. [42] Australia	To explore documentation of physiological observations by nurses in acute care Prospective observational study in one public hospital	178 patients of ward units and emergency department Physiological observations in the preceding 24 h (ward patients) or 8 h (emergency department)	(i) The most documented vital signs were RR, SpO_2_, HR, and SBP while the least documented were T° and conscious state (ii) There was evidence of one or more abnormal physiological parameters in 79.8% (142/178) of patients with documented abnormalities in only 19.7% of them (28/142)

2016 Smith and Aitken [43] UK	To investigate the use of a single-parameter TTS for implementation of the NEWS tool by nurses. To report the characteristics of patients and triggers. To explore barriers and facilitators to patient monitoring Mixed methods study in one university hospital	263 physiological triggers of 74 patients from general wards Cross-sectional survey of 105 nurses Barriers and facilitators to monitoring a deteriorating patient with a single-parameter TTS Nursing staff perceptions of the TTS	(i) The most recorded physiological trigger was the SBP (59%, 156/263) and the least recorded was the RR (14%, 36/263) (ii) Barriers and facilitators to monitor and escalate abnormal vital signs of patients were as follows: (a) Lack of equipment for vital signs monitoring (equipment) (b) Barriers to both effective monitoring of patients and the escalation process (workload) (c) Conflicting priorities between different members of the nursing staff (interactions between the staff) (d) Patients that may not consent to record observations (interactions with patients)

MET: medical emergency team; ICU: intensive care unit; CAT: cardiac arrest team; min: minutes; BP: blood pressure; HR: heart rate; RR: respiratory rate; T°: temperature; SpO_2_: peripheral oxygen saturation; SBP: systolic blood pressure; h: hours; MEWS: modified early warning score; SAEs: serious adverse events; GCS: Glasgow Coma Scale; BLS: basic life support; ALS: advanced life support; ALF: afferent limb failure; RRS: rapid response system; TTS: track and trigger system; NEWS: national early warning score.

**Table 2:** Summary of relevant studies on recognizing deteriorating patients.

Year Authors Country	Aim Design	Sample Outcome measures	Findings
2010 Donohue and Endacott [44] UK	To examine ward nurses and critical care outreach staff perceptions in acute wards Semistructured interviews with hospital clinicians	11 nurses and 3 members of the outreach team Staff perceptions in management of deteriorating patients	(i) The MEWS was not a key component of the patient assessment and was used to quantify deterioration after recognition of the patient's instability (ii) Clinicians needed better understanding of the value of TTSs in identifying trends in the patient's condition

2011 Ludikhuize et al. [45] Netherlands	To evaluate whether nurses trained in the use of the MEWS and SBAR communication tool were more effective to recognize a deteriorating patient Prospective, quasi–experimental simulation study in one teaching hospital	Simulated case study presented to 47 trained and 48 nontrained nurses The case was a fictitious deteriorating patient with the nursing chart including vital parameters Monitoring of vital signs (HR, RR, SBP, SpO_2_, and T°)	(i) The MEWS was correctly determined by 11% (4/47) of the trained nurses with better notification to the physician; the SBAR communication tool was used by only 1 nurse (ii) 77% (36/47) of the trained nurses vs. 58% (28/48) of the nontrained group assessed the patient immediately (*p*=0.056) (iii) The RR was measured twice as frequently (53% trained vs. 25% nontrained nurses, *p*=0.025) with no differences in other vital parameters

2015 Kolic et al. [46] UK	To assess scoring accuracy and adequacy of clinical responses to the NEWS, and the impact of time of day, the day of the week, and score severity on responses Prospective observational study in one general hospital	370 adult patients in an acute medical ward Two outcomes: (1) scoring errors and adequacy of clinical responses; (2) whether inadequate NEWS responses were associated with increased patient mortality	(i) The NEWS was calculated incorrectly in 18.9% (70/370) of patients with a substantial increase in scoring errors as the NEWS increased (ii) 25.9% (96/370) of patients had an inadequate responses to the NEWS (iii) Substantially worse clinical responses on weekends

2016 van Galen et al. [47] Netherlands	To perform a root-cause analysis of unplanned ICU admissions. To assess adherence to the MEWS system in identifying deteriorating patients transferred to the ICU Retrospective observational study in one university hospital	Out of 49 adult patients, 477 vital parameter sets were found in the 48 hours before ICU admission from a general ward Causes of unplanned ICU admissions and adherence to the MEWS	(i) The MEWS was calculated correctly in only 1% (6/477) of measurements, 48 h before ICU admission, although 43% (207/477) had a critical score (MEWS score ≥3) (ii) In 41% of the patients, vital signs monitoring was done as discussed with the physicians (iii) The root causes were work-related (45%), mainly failures in patient monitoring, disease-related (46%), patient-related (7.5%), and organizational-related (3%)

2017 Petersen et al. [48] Denmark	To identify barriers and facilitating factors related to the use of the EWS escalation protocol among nurses Focus group in one tertiary hospital	18 nurses Content analysis for three aspects of the EWS protocol: (1) adherence to the monitoring frequency; (2) call for junior doctors to patients with an elevated EWS; (3) call for the MET	(i) Monitoring less frequently than prescribed occurred regularly during busy periods and at night (ii) To inform doctors about patients with EWS ≥3 is not particularly important for the number of patients with an elevated score (iii) There were barriers to MET calls since many nurses had negative feelings toward the MET

2017 Wong et al. [49] Canada	To evaluate (1) how many patients had critical messages before the ICU transfer and the quality of messages; (2) whether the quality of the message, the quality of the response or the timeliness of RRT activation were related to death Retrospective observational study in one tertiary hospital	236 general ward patients All CM communicating deterioration in the 48 h before the ICU transfer CM: messages with information that met the calling criteria of the institution	(i) 39% (93/236) of patients had CM 48 h before the ICU transfer (ii) Only 45% of messages contained 2 or more vital signs and 3% contained the SBAR tool (iii) The message quality, mainly the use of the SBAR tool, was positively related to in-hospital survival

EWS: early warning score; TTS: track and trigger system; HR: heart rate; RR: respiratory rate; SBP: systolic blood pressure; T°: temperature; SpO_2_: peripheral oxygen saturation; MEWS: modified early warning score; SBAR: situation-background-assessment-recommendation; ICU: intensive care unit; NEWS: national early warning score; MET: medical emergency team; RRT: rapid response team; CM: critical messages; h: hours.

**Table 3:** Summary of relevant studies on escalating care to deteriorating patients.

Year Authors Country	Aim Design	Sample Outcome measures	Findings
2006 Jones et al. [50] Australia	To assess the attitudes of nurses to the MET system 4 years after its introduction and obstacles to its use Prospective observational survey in one university hospital	351 ward nurses Barriers to calling the MET Nurses' attitudes toward the MET system	(i) Major barriers to MET activation were the traditional model of calling a junior doctor before the MET (72%) and underestimation of physiological perturbations associated with the presence of MET call criteria (ii) Nurses would make a MET call for a patient they were worried even if the vital signs were normal (56%)

2008 Schmid-Mazzoccoli et al. [51] USA	To identify nurse, patient, and organizational variables that predict delayed MET calls Prospective observational study in one university hospital	Convenience sample of 108 MET interventions on medical and surgical general wards Delayed MET calls: MET criteria present for >30 min before the call	(i) Delayed events were 44% (47/108) often on the night shift (*p*=0.012) (ii) The shift and patient-unit-match (medical, surgical) were significant predictors of delays (iii) Patient, nurse, and organizational characteristics influenced the timely rescue

2010 Bagshaw et al. [52] Canada	To evaluate the vision of nurses on the MET system 3 years after its implementation Cross-sectional survey in one academic hospital	275 ward nurses Beliefs and behaviors of nurses regarding the MET system	(i) Nurses would call the attending physician before activating the MET (75.9%), they would activate the MET for a patient they were worried even if the patient had normal vital signs (48%), and they were reluctant to activate the MET for the fear of criticism (15.4%)

2010 Calzavacca et al. [53] Australia	To test the impact of RRS maturation on delayed MET activation (MET criterion documented at least 1 h before MET activation) and patient outcomes Before-and-after observational study in one tertiary hospital	MET reviews in a recent cohort (200 patients) and in a control cohort (400 patients) 5 years earlier of RRS implementation ICU admission, hospital LOS, and hospital mortality after MET reviews	(i) Fewer patients (22% vs. 40.3%, *p* < 0.001) had delayed MET activation in a recent cohort vs. a control cohort (ii) Delayed activation was associated with greater risk of unplanned ICU admission and hospital mortality (OR 1.79, 95% CI 1.33–2.93, *p*=0.003 and OR 2.18, 95% CI 1.42–3.33, *p* < 0.001, respectively)

2011 Trinkle and Flabouris [54] Australia	To measure and describe ALF and its impact on patient outcomes Retrospective observational study in one university-affiliated hospital	443 patients and 575 adverse events (6.1% (35/575) cardiac arrests, 68.7% (395/575) MET calls, and 25.2% (145/575) unanticipated ICU admissions) ALF as the RRS performance and the impact on patient outcomes	(i) Documented ALF was described in 22.8% (131/575) of adverse events (ii) Patients with ALF vs. those without ALF had more unanticipated ICU admissions, 34.4% (45/131) vs. 22.5% (100/444), (*p*=0.01) and higher hospital mortality across multiple, compared to single, time periods, 52.5% (21/40) vs. 31.9% (22/69), (*p*=0.03)

2012 Shearer et al. [55] Australia	To explore the causes of the failure of RRS activation in the acute adult population Multimethod study: the missed RRS incidence, the prospective study of missed RRS calls, and staff interviews in four university tertiary hospitals	570 adult observation charts, 91 staff interviews (physicians, nurses, MET members, ICU teams) involved in missed RRS calls Physiological instability and outcomes of ward patients Missed RRS calls Staff interviews	(i) 4.04% (23/570) of patients had a clinical instability, 42% of them did not receive an appropriate clinical response, although the staff recognized criteria for RRS activation (69.2%), and being “quite” or “very” concerned about their patient (75.8%) (ii) Missed RRS calls were 43.47% (10/23), the main reason was to feel that the situation was under control in the ward (51.8%) (iii) The failure to RRS activation was due to dominantly sociocultural reasons

2014 Boniatti et al. [56] Brazil	To evaluate an association between delayed MET calls and mortality Prospective observational study in one university-affiliated tertiary hospital	1,481 calls for 1,148 patients Delayed MET calls (namely documented MET criteria with no MET calls for 30 min to 24 h before a MET review) and mortality	(i) Delayed MET calls resulted in 21.4% (246/1,148) of patients, significantly higher for physicians (110/377, 29.2%) vs. nurses (136/771, 17.6%), *p* < 0.001 (ii) 30-day mortality after the MET review was higher for patients with delayed vs. timely MET activation, 61.8% (152/246) vs. 41.9% (378/902), *p* < 0.001, respectively (iii) In patients without delays, the main trigger was concern about the patient

2014 Davies et al. [57] USA	To identify barriers to activation of the RRS by clinical staff Cross-sectional survey in one tertiary hospital	68 physicians and 16 nurses on medical and surgical wards Adherence to six calling criteria: HR, MAP, RR, SpO_2_, mental status change, and “not” looking right'	(i) The self-reported adherence rate for the six activation criteria of the RRS was ≤25% (ii) The staff members were most familiar with mental status change (76.2%) and least familiar with “not looking right” (65.5%)

2015 Chen et al. [58] Australia	To test the hypothesis that delayed team calls for deteriorating ward patients were associated with increased mortality Post hoc analysis of MERIT study in 23 hospitals	3,135 emergency team calls with CAT or MET activation Patients with delayed activation (any call occurred >15 min after documented MET calling criteria) and hospital outcomes (mortality, unplanned ICU admissions, and cardiac arrests)	(i) In all hospitals, 30.2% (947/3,135) of patients had delayed calls (ii) In the MET hospitals, the proportion of delayed calls was similar before and after implementation of the RRS (iii) In all hospitals, delayed calls increased the risk of unplanned ICU admissions (adjusted OR 1.56, 95% CI 1.23–2.04, *p* ≤ 0.001) and death (adjusted OR 1.79, 95% CI 1.43–2.27, *p* < 0.001)

2015 Radeschi et al. [59] Italy	To identify attitudes toward the MET and barriers to its utilization among ward nurses and physicians Cross-sectional quantitative survey in 10 hospitals	1,812 ward nurses and physicians in hospitals with a fully operational MET system Attitudes toward the MET and barriers to its utilization	(i) Major barriers to MET activation were (1) nurse referral to the covering physician for deteriorating patients (62%); (2) the reluctance to call the MET in a patient fulfilling the calling criteria (21%) less likely in medical doctors vs. nurses, unaffected by the METal certification (ii) Medical status, working in surgical vs. medical wards, seniority, and participation in the METal training course were associated with lower likelihood of showing barriers to MET activation

2016 Barwise et al. [60] USA	To identify delays in RRT activation in hospital Retrospective observational cohort study in one tertiary academic hospital	1,725 patients and vital signs 24 h before RRT activation RRT activation and hospital patient outcomes (mortality and morbidity) Delayed activation: 1 h between the first abnormal vital sign and RRT activation	(i) 57% (977/1,725) of patients had delayed RRT activation (ii) The delayed group had higher hospital mortality (15% vs. 8%, adjusted OR 1.6, *p*=0.005), 30-day mortality (20% vs. 13%, adjusted OR 1.4, *p*=0.02), and hospital LOS (7 vs. 6 days, relative prolongation 1.10, *p*=0.02) vs. the no-delay group

2016 Castano-Avila et al. [61] Spain	To assess differences between ward patients with persistent clinical deterioration admitted to the ICU and those admitted at an earlier stage of deterioration Retrospective observational study in one tertiary university hospital	80 ICU admissions of 69 patients from hospital wards Delayed alert: ≥2 warning signs in SBP or SpO_2_ assessments, 8–24 h before ICU admissions Admissions to the ICU after delayed alerts	(i) There was a delayed alert in 41.25% (33/80) of ICU admissions. These patients had a higher APACHE II (*p*=0.001) score, SAPS II (*p*=0.01) score, MODS incidence (*p* < 0.0001) statistically significant, and nonsignificant longer ICU stays (*p*=0.052) (ii) Alerts were most frequently circulatory (33.7%) or respiratory (30%) related and realized by physicians on duty (85.2%)

2017 Gupta et al. [62] Australia	To investigate the impact of delayed RRC activation on patient outcomes Retrospective observational study in one tertiary hospital	826 RRCs across 629 admissions Delayed call: RRC activation delayed by ≥15 min In-hospital mortality, hospital LOS, and ICU admission	(i) Delayed RRCs were 24.6% (203/826) (ii) Patients with a delayed RRC had significantly higher in-hospital mortality (34.7% vs. 21.2%, *p*=0.001) and longer hospitalizations (11.6 vs. 8.4 days, *p*=0.036)

2017 Sprogis et al. [63] Australia	To investigate the frequency, characteristics, and timing of the limitation of the clinical instability 24 h before MET activation Retrospective observational study in one tertiary teaching hospital	200 adult ward patients UCR criteria breached 24 h before MET activation and in-hospital mortality	(i) 78.5% (157/200) of patients had UCR criteria at least once 24 h before MET activation. In 136/157 (86.6%) of first UCR criteria breaches no documentation was found, and in 91/157 (58%) of them there were no documented nursing actions (ii) There were suboptimal medical reviews despite activation (iii) Hospital mortality in patients after MET activation was 12%

MET: medical emergency team; RRS: rapid response system; min: minutes; ICU: intensive care unit; LOS: length of stay; OR: odds ratio; h: hours; ALF: afferent limb failure; HR: heart rate; MAP: mean artery pressure; RR: respiratory rate; SpO_2_: peripheral oxygen saturation; MERIT: medical early response, intervention, and therapy; RRT: rapid response team; METal: medical emergency team alert; SBP: systolic blood pressure; APACHE II: acute physiologic assessment and chronic health evaluation; SAPS II: simplified acute physiology score; MODS: multiple organ dysfunction syndrome; RRC: rapid response call; UCR: urgent clinical review.
