# Supplementary material for: Performance of the Afferent Limb of Rapid Response Systems in Managing Deteriorating Patients: A Systematic Review
Source: Crit Care Res Pract. 2019 Oct 30;2019:6902420. doi: 10.1155/2019/6902420 (PMC6874970; doi:10.1155/2019/6902420)
Supplement: Supplementary Materials — Supplementary data associated with this article can be found in Tables A, B, C, and D. [file 6902420.f1.pdf]

Appendix

Table A. PRISMA checklist.

Section/topic		Checklist item	Reported on page *
**TITLE**			
Title	1	Identify the report as a systematic review, meta-analysis, or both.	1
**ABSTRACT**			
Structured summary	2	Provide a structured summary, including, as applicable: background; objectives; data sources; study eligibility criteria, participants, and interventions; study appraisal and synthesis methods; results; limitations; conclusions and implications of key findings; systematic review registration number.	2
**INTRODUCTION**			
Rationale	3	Describe the rationale for the review in the context of what is already known.	4
Objectives	4	Provide an explicit statement of the questions being addressed with reference to participants, interventions, comparisons, outcomes, and study design (PICOS).	6<br>Figure 1
**METHODS**			
Protocol and registration	5	Indicate if a review protocol exists, if and where it can be accessed (e.g., Web address), and, if available, provide registration information including registration number.	6<br>Appendix (Table A)
Eligibility criteria	6	Specify study characteristics (e.g., PICOS, length of follow-up) and report characteristics (e.g., years considered, language, publication status) used as criteria for eligibility, giving rationale.	6
Information sources	7	Describe all information sources (e.g., databases with dates of coverage, contact with study authors to identify additional studies) in the search and date last searched.	6<br>Appendix (Table B)
Search	8	Present full electronic search strategy for at least one database, including any limits used, such that it could be repeated.	7<br>Appendix (Table C)
Study selection	9	State the process for selecting studies (i.e., screening, eligibility, included in the systematic review, and, if applicable, included in the meta-analysis).	7
Data collection process	10	Describe the method of data extraction from reports (e.g., piloted forms, independently, in duplicate) and any processes for obtaining and confirming data from investigators.	9
Data items	11	List and define all variables for which data were sought (e.g., PICOS, funding sources) and any assumptions and simplifications made.	9
Risk of bias in individual studies	12	Describe methods used for assessing the risk of bias of individual studies (including specification of whether this was done at the study or outcome level) and how this information is to be used in any data synthesis.	9
Summary measures	13	State the principal summary measures (e.g., risk ratio, difference in means).	N/a

Synthesis of results	14	Describe the methods of handling data and combining the results of studies, if done, including measures of consistency (e.g., I2) for each meta-analysis.	N/a
Risk of bias across studies	15	Specify any assessment of the risk of bias that may affect the cumulative evidence (e.g., publication bias, selective reporting within studies).	N/a
Additional analyses	16	Describe methods of additional analyses (e.g., sensitivity or subgroup analyses, meta-regression), if done, indicating which were pre-specified.	N/a
		**RESULTS**	
Study selection	17	Give numbers of studies screened, assessed for eligibility, and included in the review, with reasons for exclusions at each stage, ideally with a flow diagram.	8<br>Figure 2
Study characteristics	18	For each study, to present characteristics for which data were extracted (e.g., study size, PICOS, follow-up period) and to provide the citations.	9<br>Appendix (Table D)
Risk of bias within studies	19	Present data on the risk of bias of each study and, if available, any outcome-level assessment (see item 12).	9
Results of individual studies	20	For all outcomes considered (benefits or harms), present, for each study: (a) simple summary data for each intervention group (b) effect estimates and confidence intervals, ideally with a forest plot.	9<br>Table 1-3
Synthesis of results	21	Present results of each meta-analysis done, including confidence intervals and measures of consistency.	10
Risk of bias across studies	22	Present results of any assessment of the risk of bias across studies (see Item 15).	N/a
Additional analysis	23	Give the results of additional analyses, if done (e.g., sensitivity or subgroup analyses, meta-regression [see Item 16]).	N/a
		**DISCUSSION**	
mary of evidence	24	Summarize the main findings, including the strength of evidence for each main outcome; consider their relevance to key groups (e.g., healthcare providers, users, and policy makers).	15
Limitations	25	Discuss limitations at study and outcome level (e.g., risk of bias), and at review-level (e.g., incomplete retrieval of identified research, reporting bias).	20
Conclusions	26	Provide a general interpretation of the results in the context of other evidence and implications for future research.	21
		**FUNDING**	
Funding	27	Describe sources of funding for the systematic review and other support (e.g., supply of data); role of funders for the systematic review.	N/a

From: Moher D, Liberati A, Tetzlaff J, et al., PRISMA Group. Preferred reporting items for systematic reviews and meta-analyses: the PRISMA statement. BMJ. 2009 Jul 21;339:b2535; https://doi.org/10.1136/bmj.b2535. For more information, visit **www.prisma-statement.org**.

* Pages correspond to the version submitted to the journal.

Table B. SPIDER framework.

SPIDER	Eligibility criteria
**Sample**	Adult patients and ward physicians and nurses.<br>General wards of the acute hospital.
**Phenomenon of Interest**	Factors that influence, by promoting or preventing, the performance of the afferent limb of the rapid response system (RRS) in managing deteriorating patients in general wards. Primary peer-reviewed research articles in the English language, the only full text, published between January 1995 and December 2017 were included. The year 1995 was chosen for the Australian study [Reference 1] that first outlined the concept of the RRS as a team of critical care clinicians responding to deteriorating patients outside the intensive care unit.
**Design**	The designs of the included studies were randomized controlled trial, quasi-experimental study, before-and-after study, retrospective observational study, prospective observational study, cross-sectional survey, post-hoc analysis, qualitative study, and mixed methods study.
**Evaluation**	Selected studies were grouped into three domains and common areas among studies were structured into themes related to the review purpose.<br>Themes on monitoring deteriorating patients comprised lack of recording, poor documentation of respiratory rate, and influence of facilitator and barriers (effects of RRS implementation, effects of educational programs, and effects of standardized measurements and interfering factors).<br>Themes on recognizing deteriorating patients comprised compliance with the calling criteria and impact of communication between ward clinicians.<br>Themes on escalating care to deteriorating patients comprised influence of cultural barriers and personal judgment on response activation, delayed team calls, and effects of delays on clinical outcomes.
**Research type**	Research types were qualitative, quantitative, and mixed methods.

SPIDER tool (Cooke et al., 2012) is an adaptation of the PICO components to make them more suitable for qualitative and qualitative research [Reference 29].

Table C. Search strategy for CINAHL and MEDLINE.

Database CINAHL (Cumulative Index to Nursing and Allied Health Literature)
Database: CINAHL Plus with Full Text.
Interface: EBSCOhost research databases.
Limiters: Abstract available. Published Date: 1995/01/01-2017/12/31. English language. Peer reviewed. Narrow by subject age: all adults. Search modes: Boolean/Phrase and SmartText Searching.

S1	Deteriorating patients	185
S2	Rapid response systems	48
S3	Medical emergency team OR rapid response team OR critical care outreach service OR critical care response team	218
S4	Patient monitoring, patient recognizing, escalation of care, general wards	819
S5	S1 AND S2 AND S3 AND S4	837

Medline
Database: MEDLINE.
Interface: EBSCOhost research databases.
Limiters: Abstract available. Published Date: 1995/01/01-2017/12/31. English language. Peer reviewed. Narrow by subject age: all adults. Search modes: Boolean/Phrase and SmartText Searching.

S1	Deteriorating patients	659
S2	Rapid response systems	128
S3	Medical emergency team OR rapid response team OR critical care outreach service OR critical care response team	557
S4	Patient monitoring, patient recognizing, escalation of care, general wards	3,462
S5	S1 AND S2 AND S3 AND S4	4,968

Table D. Summary of key study characteristics.

**Country**	▪ Australia (n = 13)<br>▪ Netherlands (n = 4)<br>▪ United Kingdom (n = 3) and United States of America (n = 3)<br>▪ Canada (n = 2)<br>▪ Brazil (n = 1), Denmark (n = 1), Finland (n = 1), Greece (n = 1), Italy (n = 1), and Spain (n = 1)
**Setting**	▪ Community, teaching, and university hospitals (n = 30)<br>▪ Simulation scenario (n = 1)
**Sample size**	▪ Range (min-max): 14*-125,132**
**Population**	▪ General ward patients (n = 22)<br>▪ Ward nurses (n = 7)<br>▪ Ward physicians and nurses (n = 2)
**Designs of studies**	▪ Cluster randomized controlled trial (n = 1)<br>▪ Quasi-experimental study (n =1)<br>▪ Before-and-after study (n = 1)<br>▪ Retrospective observational study (n = 9)<br>▪ Prospective observational study (n = 9)<br>▪ Cross-sectional survey (n = 4)<br>▪ Post-hoc analysis (n = 2)<br>▪ Qualitative study (n = 2)<br>▪ Mixed methods study (n = 2)
**Findings of selected studies**	▪ Monitoring deteriorating patients (n = 11)<br>▪ Recognizing deteriorating patients (n = 6)<br>▪ Escalating care to deteriorating patients (n = 14)

[Reference **33, *44]
